# Supplementary material for: The interactome of intact mitochondria by cross-linking mass spectrometry provides evidence for coexisting respiratory supercomplexes
Source: Mol Cell Proteomics. 2017 Dec 8;17(2):216–32. doi: 10.1074/mcp.RA117.000470 (PMC5795388; doi:10.1074/mcp.RA117.000470)
Supplement: Supplemental Data [file supp_RA117.000470_133922_0_supp_23978_fzffwf.zip › spectra_annotation/mito_DR_spectra_annotation/455-1-12-1-10-1.pdf]

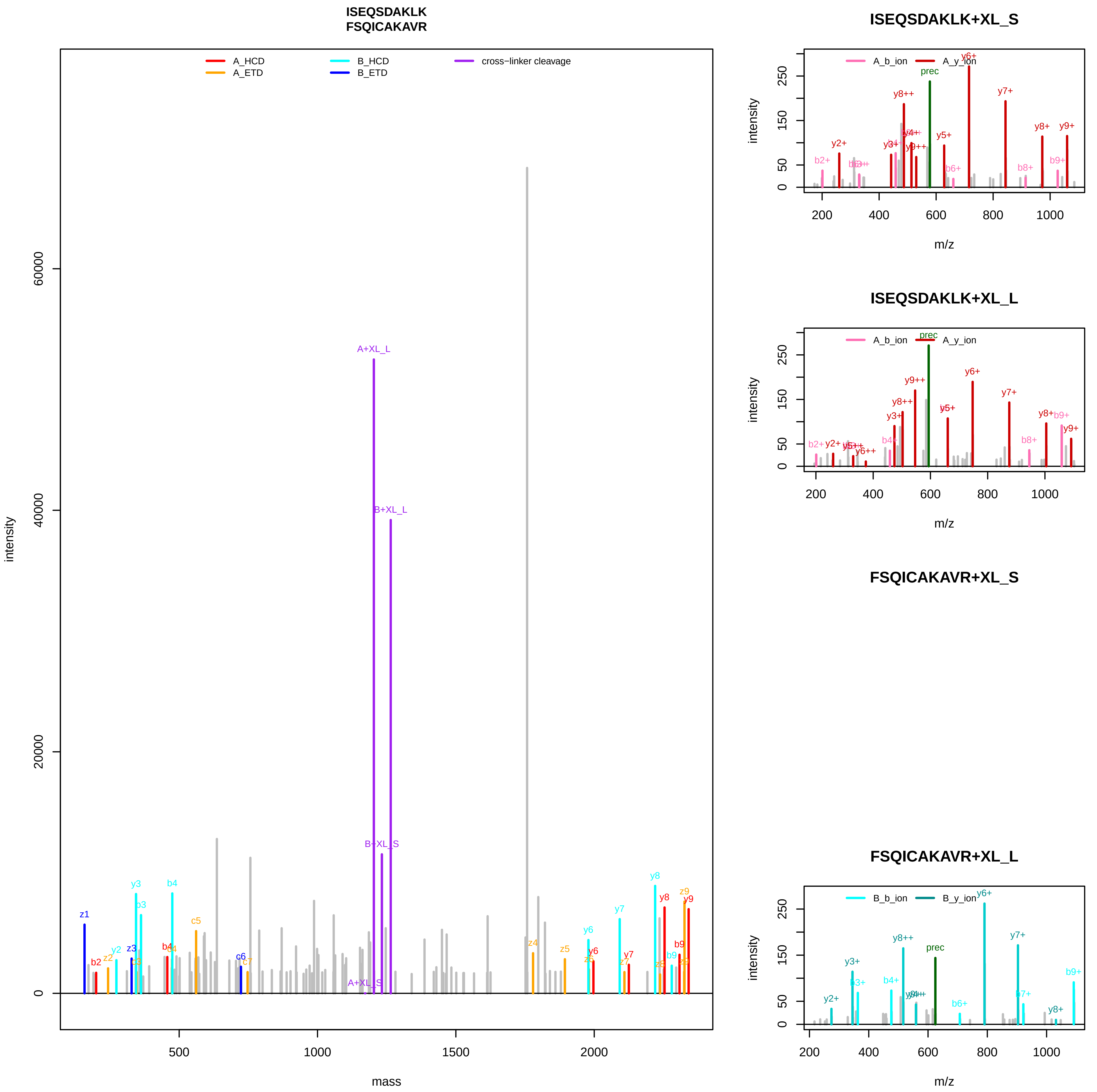
ISEQSDAKLK
FSQICAKAVR
A_HCD
A_ETD
B_HCD
B_ETD
cross−linker cleavage
intensity
mass
0
20000
40000
60000
500
1000
1500
2000
A+XL_L
B+XL_L
B+XL_S
A+XL_S
z1
b2
z2
y2
z3
y3
b3
b4
c4
c5
c6
c7
z4
z5
z6
y6
y7
z7
y8
z8
b9
z9
y9
ISEQSDAKLK+XL_S
ISEQSDAKLK+XL_L
FSQICAKAVR+XL_S
FSQICAKAVR+XL_L
A_b_ion
A_y_ion
B_b_ion
B_y_ion
prec
m/z
200
400
600
800
1000
0
50
150
250
y2+
y3+
y5+
y6+
y7+
y8+
y9+
y8++
y9++
b2+
b4+
b6+
b8+
b9+
